# Ultrasound-Mediated Bioeffects in Senescent Mice and Alzheimer’s Mouse Models

**DOI:** 10.3390/brainsci12060775

**Published:** 2022-06-13

**Authors:** Matilde Balbi, Daniel G. Blackmore, Pranesh Padmanabhan, Jürgen Götz

**Affiliations:** 1 Queensland Brain Institute, The University of Queensland, Brisbane, QLD 4072, Australia; m.balbi@uq.edu.au (M.B.); d.blackmore@uq.edu.au (D.G.B.); p.padmanabhan@uq.edu.au (P.P.); 2 Clem Jones Centre for Ageing Dementia Research, Queensland Brain Institute, The University of Queensland, Brisbane, QLD 4072, Australia

**Keywords:** amyloid, long-term potentiation, low-intensity ultrasound, mechanosensory receptor, neurogenesis, neuromodulation, NMDA receptor (NR), senescence, Tau

## Abstract

Ultrasound is routinely used for a wide range of diagnostic imaging applications. However, given that ultrasound can operate over a wide range of parameters that can all be modulated, its applicability extends far beyond the bioimaging field. In fact, the modality has emerged as a hybrid technology that effectively assists drug delivery by transiently opening the blood–brain barrier (BBB) when combined with intravenously injected microbubbles, and facilitates neuromodulation. Studies in aged mice contributed to an insight into how low-intensity ultrasound brings about its neuromodulatory effects, including increased synaptic plasticity and improved cognitive functions, with a potential role for neurogenesis and the modulation of NMDA receptor-mediated neuronal signalling. This work is complemented by studies in mouse models of Alzheimer’s disease (AD), a form of pathological ageing. Here, ultrasound was mainly employed as a BBB-opening tool that clears protein aggregates via microglial activation and neuronal autophagy, thereby restoring cognition. We discuss the currently available ultrasound approaches and how studies in senescent mice are relevant for AD and can accelerate the application of low-intensity ultrasound in the clinic.

## 1. Introduction to Therapeutic Ultrasound

In this hybrid between an opinion piece and a review article, we present the blood–brain barrier (BBB) as the major impediment in developing effective disease-modifying treatments for psychiatric and neurological conditions such as Alzheimer’s disease (AD). Further reasons, besides either inefficient or a complete lack of brain drug uptake and retention due to the BBB’s gatekeeper function, are a lack of robust biomarkers, an unknown aetiology for non-genetic AD cases, and often a long delay between disease-associated pathological brain changes and the onset of clinical symptoms. These reasons may explain why recent clinical trials involving therapeutics targeting the core pathologies of AD have reported a lack of association between the clearance of protein deposits such as amyloid-β (Aβ) plaques (a hallmark lesion of AD) and a slowing in cognitive decline [1]. It is therefore not surprising that non-pharmacological approaches such as deep brain stimulation (DBS) or transcranial magnetic stimulation (TMS) are being explored for the treatment of AD to circumvent the limitations of traditional pharmacological therapies [2,3]. A promising, emerging hybrid technology discussed here is therapeutic ultrasound (US). In addition to facilitating neuromodulation (akin to DBS or TMS), the US also effectively assists drug delivery by transiently opening the BBB when combined with intravenously injected microbubbles that are induced to oscillate and thereby cause pressure on the BBB’s tight junctions that transiently open and make the interstitial space of the brain accessible [4].

US is a mechanical pressure wave (sound) at a frequency above the range of human hearing (>20 kHz), and it is used routinely in the MHz range for diagnostic imaging applications. For therapeutic purposes, US is applied to the human brain at much lower frequencies (typically <500 kHz) than for imaging purposes aimed at, e.g., peripheral soft tissue such as the womb. This is because high-frequency US is attenuated by the human (and less so by the mouse) skull. When delivered through a single curved transducer (or an array composed of either flat or curved transducers), the ultrasound energy can be most effectively focused on the target within the brain tissue (FUS). Of note, therapeutic US possesses numerous advantages over more traditional approaches. For example, unlike conventional drug treatment, directing the US beam exclusively to distinct brain areas, such as the hippocampus, allows for both region-specific and brain-wide treatment. The former is critical for more confined diseases, such as Parkinson’s disease, with spatially restricted pathology, and the latter, we would argue, for diseases with a more diffuse and wide-spread pathology such as AD. In addition, in contrast to radiation therapy, US exerts its effects only in the focal zone and not in the tissue through which the sound waves travel, thereby enhancing its safety profile.

The FDA has approved high frequency US applied as a continuous (i.e., unpulsed) waveform to coagulate (ablate) thalamic tissue (thalamotomy), for the treatment of essential tremor (ET) and tremor-dominant Parkinson’s disease (TDPD) [5,6]. Other FUS mechanisms under active investigation, several in the context of brain tumours, are hyperthermia (to induce uptake of chemotherapeutics and immunogenicity [7]), sonodynamic therapy (using US to generate reactive oxygen species from sonosensitisers [8]), histotripsy (to liquefy blood clots), and sonothrombolysis (to remove blood clots in stroke patients [9,10]), as recently discussed in detail in an excellent review [11].

By using pulsed waveforms at low sub-MHz frequencies, US has further been explored as a non-invasive tool to either open the BBB and/or induce neuromodulatory effects. This division is in some respect operational because in order to induce BBB opening with US, this does not necessarily exclude US also exerting neuromodulatory effects. We refer the reader to several excellent reviews that are available in the neuromodulatory space using US [12,13,14,15,16].

**Figure 1:**
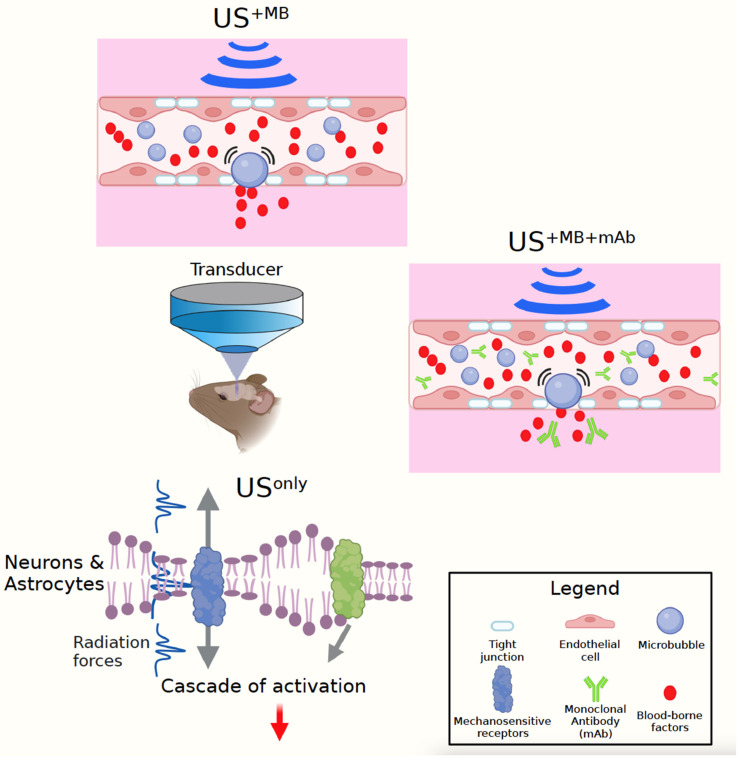
**Ultrasound modalities:** This review discusses three ultrasound (US) modalities, with a transducer generating the sound waves which travel through the skull into the mouse brain. To open the blood–brain barrier (BBB), microbubbles (MBs) are intravenously injected (US^+MB^). Upon US exposure, the MBs start to oscillate (or cavitate) in the brain capillaries, thereby causing pressure on the BBB’s tight junctions that transiently open and make the interstitial space of the brain accessible (US^+MB^). This modality relies on the therapeutic effects of endogenous, unidentified blood-borne factors that are taken up by the brain. US^+MB+mAb^ uses therapeutic agents as exemplified by monoclonal antibodies (mAbs) that together with blood-borne factors are taken up by the brain when the BBB opens within the focal volume of the US beam. Finally, US can also be used on its own, in the absence of BBB opening (US^only^). The principal mechanisms of US are the generation of heat (for the work discussed here largely negligible), its radiation force, and cavitation, with the latter coming into play, in particular, with the use of MBs in the two modalities US^+MB^ and US^+MB+mAb^. In US^only^, in the absence of significant cavitation and heating, the dominating principle is the radiation force (together with the rapid expansion/contraction of neuronal membranes). This is shared between the three US modalities and induces a cascade of activation events in cell types such as astrocytes and neurons, as discussed here. Figure generated with BioRender.

Another way to look at US is to subdivide this modality into three fundamental approaches, US^only^, US^+MB^, and US^+MB+mAb^ (Figure 1). The first approach uses low-intensity US on its own. The second uses low-intensity US together with intravenously injected microbubbles (MBs) to transiently open the BBB, relying on the therapeutic effects of endogenous, unidentified blood-borne factors that are taken up by the brain. The third uses in addition therapeutic agents, such as monoclonal antibodies (as discussed here), which together with blood-borne factors are taken up by the brain when the BBB opens within the focal volume of the US beam [17]. It is essential to realise that in rodents these experiments are generally performed under anaesthesia (with ketamine presenting a potential confound [18]), and a sham control needs to be included in that the animals are anaesthetised and injected with MBs, but without ultrasound being delivered. A second sham control that can be included, depending on the type of anaesthesia, are untreated and unanaesthetised (naïve) mice [19].

Yet another way to look at US is by focusing on the principal mechanisms of how this modality creates its effects: the generation of heat, radiation force, cavitation, and the rapid expansion/contraction of neuronal membranes (which is somewhat of a cross between intracellular ‘cavitation’ and heating [20]) (Figure 1). The choice of US parameters (frequency, acoustic pressure, pulse repetition frequency, duty cycle, pulse length, sonication duration, and microbubble application) dictates the extent to which the temperature in the target tissue, or in the skull through which the sound waves travel, rises: how much cavitation (i.e., bubble formation) is induced and how strong the mechanical radiation force is. Cavitation, typically measured with a passive cavitation detector (PCD), occurs when naturally occurring gas-filled bubbles inside biological tissue expand and contract or even collapse in response to the applied acoustic pressure [21]. The mechanical index (given by MI = PNP⁄√f, where PNP is the peak negative pressure and *f* is the frequency) is used to gauge the likelihood of cavitation. When bubbles are administered exogenously, e.g., by intravenously injecting preformed microbubbles (contrast agents), the cavitation effect is augmented, and the BBB can be transiently opened in a safe manner.

For the US^only^ paradigm, which does not achieve BBB opening, the bioeffects are therefore purely neuromodulatory, caused by the US’s radiation force, whilst thermal effects and cavitation are negligible (Figure 1). The radiation force initiates a cascade of events, predominantly via the activation of mechanosensitive receptors as discussed below [22,23]. The radiation force in this manner also causes the contraction and expansion of the intramembrane space of the plasma membrane of neural cells, which likely also affects mechanosensitive channels [20]. For the US^+MB^ paradigm, the underlying mechanisms include the radiation force plus cavitation of the injected MBs. It should be noted that the effects of these two forces on a given biological read-out are not always additive or synergistic. Finally, for the US^+MB+mAb^ paradigm, in addition to blood-borne factors, the monoclonal antibody is taken up by the brain and, to some extent, into its cellular constituents such as neurons [24]. However, this last step is not trivial, and the conditions for achieving a wide-spread and efficient uptake are still not readily understood. On the plus side, monoclonal antibodies have become the predominant class of new drugs developed in recent years, and in 2018, eight of the top ten bestselling drugs worldwide were biologics [25]. Combining US^+MB^ with therapeutic antibodies, which are taken up by the brain only at very low 0.1% concentrations [26], therefore presents an attractive option to explore.

## 2. Applications of Low-Frequency Ultrasound to Alzheimer’s Mouse Models

In this section, we discuss the application of US to AD mouse models. Histopathologically, AD is characterised by extracellular Aβ deposits (senile plaques), intraneuronal Tau aggregates (neurofibrillary tangles and neuropil threads), as well as neuroinflammation [27]. The currently available AD therapies (except for the recently approved—and highly disputed—anti-Aβ antibody aducanumab) do not delay the neurodegenerative process and only provide symptomatic relief. Approved AD drugs are the acetylcholine esterase inhibitors donepezil (Aricept), rivastigmine (Exelon) and galantamine (Razadyne), and the NMDA receptor antagonist memantine (Namenda). The mainstay of the clinical trials that have been performed over the recent years are vaccines targeting Aβ and Tau, based on the assumption that these two molecules, which form insoluble toxic protein aggregates that constitute the hallmark lesions of AD, both initiate and drive the disease. The leading hypothesis in the field is the amyloid cascade hypothesis that places Aβ upstream of Tau [28]; however, there is also a crucial role for Tau given that Aβ toxicity is Tau-dependent, as formulated in the Tau axis hypothesis [29,30]. In transgenic animal models with either Aβ or Tau pathology, transgenic expression of mutant forms of the genes encoding APP (from which Aβ is derived by proteolytic cleavage) and Tau leads to the formation of pathological aggregates and ensuing cognitive impairments, and removing these aggregates restores cognitive functions [31].

Regarding protein aggregates, there are two fundamental treatment options, either preventing their formation in the first place or when they have formed removing them. It seemed therefore reasonable to trial the US technology and attempt to clear Aβ and Tau aggregates and thereby restore cognitive functions. It came as a surprise that Aβ could be significantly cleared by using US^+MB^ without a therapeutic agent [32,33]. By using US in a scanning mode (SUS) and 5–8 weekly treatment sessions, not only was Aβ effectively cleared, but memory functions were also improved or even restored as shown in three complementary behavioural tests, the Y-maze, novel objection recognition, and active place avoidance tests [33]. Aβ clearance by dormant microglia was identified as an underlying therapeutic mechanism. The microglia had taken up Aβ into their lysosomes, a process potentially mediated by unknown blood-borne factors that had entered the brain in response to US^+MB^-mediated BBB opening [32,33]. In a recent RNAseq transcriptomic analysis of microglia isolated from US^+MB^-treated APP23 mice followed by incubation with the amyloid dye methoxy-XO4, ‘cell cycle’ and ‘phagocytosis’ genes were specifically activated, hinting at the underlying microglial activation mechanism [34] (see also a recent study aggregating 75 murine transcriptomes in response to US^+MB^ [35]). Importantly, US^+MB^ was even shown to be safe in aged APP23 mice that display pronounced cerebral amyloid angiopathy (CAA). Again, Aβ was found to be cleared effectively by microglia and while not reducing the total plaque area, US^+MB^ reduced the fraction of large plaques. An obvious question is whether the clearance effects are long-lasting. Poon and colleagues performed a time-course experiment using two-photon microscopy to examine this. The study revealed that one single US^+MB^ session (dose) reduced the size of plaques for two weeks, advocating fortnightly treatment sessions in human AD [36] (for comparison, vaccinations are often spaced out by four weeks). Longer time frames have not been investigated [36]. The team also performed two-photon microscopy to assess leukocyte infiltration in response to US^+MB^ treatment and found significantly more neutrophils in the sonicated compared to the contralateral hemisphere, suggesting a role not only for brain-resident microglia but also peripheral neutrophils, the first line of defence of the innate immune system [37]. Of note, US^+MB^ has assisted the delivery of other agents, including the GSK inhibitor AR-A014418 [38], a TrkA agonist [39], and exosomes [40] in Aβ-depositing mice, and Aβ-targeting metal complexes in wild-type mice [41]. More recently, stem cells have been delivered together with anti-Aβ antibodies [42]. Of note, while in APP23 mice it has been shown that BBB opening is required to clear Aβ [43], US^only^ applied in another AD mouse model (using a transducer at a higher (1.875 MHz) frequency) activated endothelial nitric oxide synthase (eNOS), an enzyme with a role in angiogenesis and vasodilation, stimulated microglia and removed Aβ [44]. In the same publication, US^only^-induced cognitive improvements in a model of vascular dementia were found to be abrogated on an eNOS knockout background [44].

US^+MB^ has also been applied to Tau transgenic mouse models. Tau is a much harder target than Aβ, given that its pathology is largely intracellular and therefore less accessible to therapeutic agents. In Tau transgenic pR5 mice (that carry the P301L mutation found in frontotemporal dementia), US^+MB^ (which constituted one of four treatment arms) reduced Tau pathology as shown for the amygdala [24]. Reduced Tau pathology was also reported in another Tau transgenic mouse model, rTg4510, using unilateral US^+MB^ treatments [45]. In Tau transgenic K3 mice (carrying the frontotemporal dementia mutation K369I) with a memory and motor deficit, US^+MB^ was shown to partly ameliorate these behavioural phenotypes, and Tau was cleared by the activation of neuronal autophagy (rather than microglial activation) [46]. Given that Tau is a protein that has been demonstrated to be degraded in lysosomes via autophagy [47], US^+MB^ therefore appears to boost an intrinsic mechanism that neurons use to clear Tau. Finally, US^+MB^ has also been applied to a mouse model with both Aβ and Tau pathology in the 3xTg-AD strain (although in these mice the Tau pathology is very modest) [48]. The study confirmed microglial engulfment of amyloid and improvements in learning and memory. Moreover, a proteomic analysis was performed to gain additional insight into potential mechanisms.

US^+MB^ has further been combined with anti-Tau and anti-Aβ antibodies, exploring antibody formats ranging from single-chain variable fragments (ScFvs) with a molecular weight of 29 kDa to full-sized immunoglobulins with a molecular weight of 150 kDa. Nisbet and colleagues explored US^+MB^ together with an ScFv antibody fragment targeting 2N Tau (RN2N), with the epitope being present in 2N4R Tau isoform over-expressing pR5 mice [24]. The study found that the combination treatment was superior to the single treatment arms of either using US^+MB^ or the RN2N ScFv alone. Interestingly, in the combination treatment arm, the antibody fragment was not only shown to be taken up by the brain, but also to effectively distribute into neuronal cell bodies and dendrites, demonstrating that US^+MB^ not only overcomes the BBB but also the plasma membrane as the second barrier [24]. In a follow-up study, different RN2N antibody formats were explored (ScFv, Fab and IgG), revealing an up to 30-fold increased uptake of the therapeutic antibody mediated by US^+MB+mAb^ [49].

Jordao and colleagues evaluated the anti-Aβ antibody BAM-10 and showed US^+MB^-mediated Aβ reductions in the TgCRND8 mouse model [50]. The clinically approved anti-Aβ antibody aducanumab has been tested in APP23 mice in a combination trial, revealing improved cognitive outcomes compared to the single treatment arms of either antibody alone or US^+MB^ alone [51]. A similar approach was pursued by Lemere and colleagues, who delivered an anti-pyroglutamylated Aβ antibody to aged APP/PS1dE9 mice [52]. They showed that mice administered with the combination treatment had reduced hippocampal plaque burden compared to controls. In contrast, in the aducanumab study, all three treatment arms (antibody alone, US^+MB^ alone, and the combination treatment US^+MB+mAb^) reduced plaque burden in the hippocampus, whereas in the cortex, only the combination treatment was effective [51]. It was argued that hippocampal plaques develop later than cortical plaques, and that the differences observed in clearance for the two brain areas could be related to the timing of the treatments.

Collectively, these promising preclinical data have spurred several small cohort clinical trials that have shown safety and feasibility of US^+MB^-mediated BBB opening in AD (NCT04118764, NCT04526262, NCT03119961, NCT03739905, NCT03671889, and NCT02986932). The trials differ in that they either use the MRI-guided ExAblate Neuro system multi-element array operated at 220 kHz (Insightec) [53,54], a 1 MHz transducer implanted in the skull bone thickness and connected to an external power supply via a transdermal needle connection during activation and facing the brain (Carthera: Sonocloud [55]), or a single 0.25 MHz element transducer with guidance provided by the Brainsight neuronavigation system (Columbia University) as outlined in a macaque study [56]. Whether US^+MB^-medicated BBB opening will achieve statistically significant Aβ reductions and cognitive improvements in human patients, and whether these improvements are long-lasting, needs to be determined. In support of combining US^+MB^ with an anti-Aβ antibody, in a recent article, Karran and De Strooper argue that ‘the speed of amyloid removal from the brain by a potential therapy will be important in demonstrating clinical benefit in the context of a clinical trial’ [1], clearly making a case for US^+MB^-mediated enhanced delivery of antibodies such as aducanumab into the brain of AD patients.

## 3. Ultrasound-Mediated Neuromodulation to Treat Dementia—Lessons from Senescent Mice

The major risk factor for AD is age, and it is therefore not surprising that the pathology in, for example, transgenic mouse models expressing mutant forms of APP and Tau progresses with age, as does the ensuing cognitive impairment [31]. However, studies using these models are generally conducted in mice of an early age, but C57Bl/6 mice have an average life span of around 26 months [57], and C57Bl/6 is the inbred strain on which most AD transgenic mice have either been generated or onto which they have been backcrossed. While this is a reasonable approach to understanding pathogenic processes, therapeutic strategies depend on whether the objective is to slow down the pathogenic process, cure the disease, or ask whether the treatment is also effective at an advanced age.

In a recent review, nine hallmarks of ageing have been highlighted: altered intercellular communication, deregulated nutrient sensing, stem cell exhaustion, telomerase attrition, genomic instability, epigenetic alterations, loss of proteostasis, cellular senescence, and mitochondrial dysfunction [58]—and without exception these hallmarks are all more severely affected in AD. However, discriminating between healthy brain ageing and changes due to AD remains a challenge [59], although several studies have revealed structural differences as well as different physiological rates and sites of atrophy compared to pathological ageing such as AD [60,61,62].

Given that cognitive functions become impaired in physiological ageing and not just AD, the question can be asked whether US (having shown efficacy in AD mouse models) would also improve cognitive functions in senescence. Twelve months represents mid-life for C57Bl/6 wild-type mice, and at this age the animals are still capable of spatial learning as revealed in the active place avoidance (APA) test [63]. This test can be performed over 5 days or just 30 min [19,33]. In this spatial learning and memory test, the mice are placed on a rotating platform containing a stationary shock zone. Using visual cues, the mice learn to (actively) avoid the aversive shock zone. Multiple parameters such as the total number of shocks, time to first or second entry, or the maximal time avoiding the shock zone are available to assess the cognitive ability of the mice. As the mice become older, their cognitive functions deteriorate [64], and by 18 months of age, their spatial learning is severely compromised [65]. The same holds true for long-term potentiation (LTP), an electrophysiological correlate of memory that can be induced at 12 and 18 but not at 20–22 months of age. Ageing further leads to a gradual decline in neurovascular coupling, which is the ability of the microcirculation to respond to neuronal activation [66]. This essential homeostatic mechanism ensures the brain’s proper function.

To our knowledge, only one study explored cognition in US-treated senescent mice [19]. Building on earlier work in young and middle-aged mice using a similar study protocol [67,68], 20–22-month-old senescent C57Bl/6 wild-type mice were treated weekly over six sessions with US^+MB^ and also US^only^ [19]. Surprisingly, both paradigms fully restored LTP induction. When spatial memory was assessed in the APA test, this revealed a trend towards improved cognition in the US^+MB^ paradigm, whereas the US^only^ treatment resulted in statistically significant improvements. Moreover, in a time-course experiment, gradual memory improvements were found with increasing numbers of US^only^ treatment sessions. Together, this suggests, akin to typical drug treatments, that spatial memory improvements in response to US follow a dose–response curve. The data suggested that US-mediated restoration in senescent mice involves the disintegration of perineuronal nets (PNNs), a highly condensed structure of the extracellular matrix in the central nervous system, as well as neurogenesis [19]. US^+MB^ has previously been shown to induce neurogenesis in wild-type mice [69] and AD mouse models [48], and to increase synapse formation [70], but these studies were all conducted in young animals. In addition, in the senescent mice, activation of the NMDA receptor signalling pathways based on SWATH proteomic mass spectrometry and Western blotting of differentially fractionated hippocampal tissue was observed [19]. We believe this is the central mechanism where digestion of PNNs and neurogenesis converge. While there were differences in the different read-outs between US^+MB^- and US^only^-treated mice, the key message is that both paradigms caused substantial functional improvements.

The question arises what exactly mediates the effects on the NMDA receptor. A recent study by Oh and colleagues may provide a possible explanation [22]. Using both in vitro and in vivo models, the study revealed that US activates the mechanosensitive Ca^2+^ channel TRPA1, a transient receptor potential (TRP) family member, in astrocytes. To unravel the role of this receptor, the team stimulated the motor cortex of wild-type and TRPA1 knockout (KO) mice in vivo with US, followed by scoring tail movement that for both genotypes was increased in an intensity-dependent manner. However, this movement was significantly reduced in TRPA1 KO compared to wild-type mice, suggesting that TRPA1 mediates US-induced neuromodulation and motor behaviour. A potential confound of an impaired auditory system was ruled out. The team then showed that TRPA1 activation in astrocytes mediates calcium entry, which in turn activates the glutamate-permeable, Ca^2+^-activated anion channel Best1, leading to the release of glutamate. This then activates neighbouring synaptic NMDA receptors, eliciting the observed neuromodulatory effect. Therefore, this study suggests that the effect of US on neurons is indirect and mediated through neuronal NMDA receptors. Given that the study in senescent mice also found increased TRPA1 levels for the US^only^ condition [19], a scheme emerges by which US induces both LTP induction and improvements in spatial memory via pleiotropic mechanisms. This includes NMDA receptor activation, mediated through an astrocyte/neuron interaction via Ca^2+^ activation and glutamate release. A recent study from Michael Shapiro’s group (albeit performed in primary neurons and not in vivo) suggests that US exerts its effects directly on neurons, as shown by neuronal excitation and synaptic transmission, by pathways ‘internally within neurons [that do] not require synaptic transmission [23] via neurons [71] or astrocytes [22]’. Of note, this study used a transducer with a centre frequency of 300 kHz, whereas throughout our work we used a 1 MHz transducer. When the Shapiro team treated neurons with the postsynaptic blockers AP5 (a selective NMDA receptor antagonist that competitively inhibits the ligand binding site of NMDA receptors) and CNQX, the neuronal response to US was unaffected, suggesting that each neuron responds to US individually. Cheng and colleagues used a transducer with a centre frequency of 30 MHz and found that it is possible to suppress neuronal activity in awake mice [72]. In the context of the NMDA receptor playing a critical role in Aβ-induced and Tau-mediated excitotoxicity in AD, the question arises how to tailor US such that, taking the findings obtained in senescent mice into an AD context, NMDA receptor-mediated physiological signalling occurs without causing toxicity due to over-excitation [29,30].

The picture that emerges is that more work needs to be conducted to resolve and understand exactly how US^only^ and US^+MB^ restore cognition in senescent mice and what can be learned for pathological ageing such as AD.

## 4. Conclusions and Outlook

As discussed above, safety has been demonstrated in a handful of US^+MB^ trials for AD. US^only^ has eventually also been explored in AD patients, using shockwaves (Neurolith, Storz Medical), with safety as primary outcome [73,74]. However, as for the US^+MB^ trials, extracting information about potential efficacy from these safety trials is challenging not only because of the inherent confounds of amyloid-PET, but also the high variability and heterogeneity in plaque load, both between patients and amongst brain areas. Moreover, these studies have been designed to have only one arm (single group or sequential assignment), i.e., they lack a placebo control, and in those instances where memory functions have been reported, a potential training effect cannot be ruled out given that memory functions have been tested multiple times in the same patient. To address this confound, a double-blind randomised placebo-controlled crossover US^only^ trial using shockwaves is currently being conducted, using performance in the CERAD test as the primary outcome (NCT03770182).

US^only^, i.e., US used as a purely neuromodulatory tool, has also been explored in conditions other than AD, including depression, disorders of consciousness, and epilepsy, as well as in healthy individuals, with primary outcome measures including safety and feasibility and, in many instances, constituting proof-of-concept studies and including, for example, EEG recordings and fMR imaging [75,76,77,78,79,80,81,82,83,84,85,86,87,88,89,90]. Importantly, most of the studies either reported no adverse events, or sensory responses.

A major challenge of establishing a unified concept in what works and what does not work lies in the fact that the effects of US on neural tissue depend on the US parameters being used [91]. Further, transducers, for example, can be focal or flat, and be used as single transducers of various shapes or arranged into any manner of arrays. Combining this with the fact that transducers can operate over a wide range of frequencies, which impacts the exerted radiation force, makes the task of comparing different studies exceptionally difficult. When US is used in combination with microbubbles, this adds yet another layer of complexity, as BBB opening (due to the uptake of blood-borne factors that have an impact on the cellular constituents and the extracellular matrix of the brain) does not occur in the absence of neuromodulatory effects of US due to its radiation force. In addition, the type of microbubble, its concentration, size distribution, and delivery method (bolus versus injection) greatly contribute to the outcome of the US^+MB^ procedure [92]. Due to differences in the cellular composition and tissue organisation of specific regions of the brain, there may also be critical differences in response to US, including varied responses for a given brain region between different species. An aged animal may react differently than a young animal, and the immune status of an animal or individual may also contribute to the observed outcomes. What would help the field, as has been suggested in a recent recommendation, is the implementation of a standardised approach of performing preclinical as well as clinical studies, and further to adopt a standardised system in reporting therapeutic US parameters (which needs to include the entire US system, the implemented quality control measures, as well as the protocols) in order to better compare the observed bioeffects across different studies [93]. Regardless of these challenges, it is exciting to see how far the US field has come and it is hoped that the technology one day will become an established clinical practice.

## Data Availability

Not applicable.

## References

[1] Karran E., De Strooper B. (2022). The amyloid hypothesis in Alzheimer disease: New insights from new therapeutics. Nat. Rev. Drug Discov..

[2] Koch G., Bonni S., Pellicciari M.C., Casula E.P., Mancini M., Esposito R., Ponzo V., Picazio S., Di Lorenzo F., Serra L. (2018). Transcranial magnetic stimulation of the precuneus enhances memory and neural activity in prodromal Alzheimer’s disease. NeuroImage.

[3] Targum S.D., Fosdick L., Drake K.E., Rosenberg P.B., Burke A.D., Wolk D.A., Foote K.D., Asaad W.F., Sabbagh M., Smith G.S. (2021). Effect of Age on Clinical Trial Outcome in Participants with Probable Alzheimer’s Disease. J. Alzheimers Dis..

[4] Burgess A., Hynynen K. (2016). Microbubble-Assisted Ultrasound for Drug Delivery in the Brain and Central Nervous System. Adv. Exp. Med. Biol..

[5] Elias W.J., Lipsman N., Ondo W.G., Ghanouni P., Kim Y.G., Lee W., Schwartz M., Hynynen K., Lozano A.M., Shah B.B. (2016). A Randomized Trial of Focused Ultrasound Thalamotomy for Essential Tremor. N. Engl. J. Med..

[6] Sinai A., Nassar M., Sprecher E., Constantinescu M., Zaaroor M., Schlesinger I. (2022). Focused Ultrasound Thalamotomy in Tremor Dominant Parkinson’s Disease: Long-Term Results. J. Parkinson’s Dis..

[7] Cohen-Inbar O., Xu Z., Sheehan J.P. (2016). Focused ultrasound-aided immunomodulation in glioblastoma multiforme: A therapeutic concept. J. Ther. Ultrasound.

[8] Yoshida M., Kobayashi H., Terasaka S., Endo S., Yamaguchi S., Motegi H., Itay R., Suzuki S., Brokman O., Shapira Y. (2019). Sonodynamic Therapy for Malignant Glioma Using 220-kHz Transcranial Magnetic Resonance Imaging-Guided Focused Ultrasound and 5-Aminolevulinic acid. Ultrasound Med. Biol..

[9] Baron C., Aubry J.F., Tanter M., Meairs S., Fink M. (2009). Simulation of intracranial acoustic fields in clinical trials of sonothrombolysis. Ultrasound Med. Biol..

[10] Alexandrov A.V., Kohrmann M., Soinne L., Tsivgoulis G., Barreto A.D., Demchuk A.M., Sharma V.K., Mikulik R., Muir K.W., Brandt G. (2019). Safety and efficacy of sonothrombolysis for acute ischaemic stroke: A multicentre, double-blind, phase 3, randomised controlled trial. Lancet Neurol..

[11] Meng Y., Hynynen K., Lipsman N. (2021). Applications of focused ultrasound in the brain: From thermoablation to drug delivery. Nat. Rev. Neurol..

[12] Naor O., Krupa S., Shoham S. (2016). Ultrasonic neuromodulation. J. Neural Eng..

[13] Tyler W.J., Lani S.W., Hwang G.M. (2018). Ultrasonic modulation of neural circuit activity. Curr. Opin. Neurobiol..

[14] Munoz F., Aurup C., Konofagou E.E., Ferrera V.P. (2018). Modulation of Brain Function and Behavior by Focused Ultrasound. Curr. Behav. Neurosci. Rep..

[15] Blackmore J., Shrivastava S., Sallet J., Butler C.R., Cleveland R.O. (2019). Ultrasound Neuromodulation: A Review of Results, Mechanisms and Safety. Ultrasound Med. Biol..

[16] Rabut C., Yoo S., Hurt R.C., Jin Z., Li H., Guo H., Ling B., Shapiro M.G. (2020). Ultrasound Technologies for Imaging and Modulating Neural Activity. Neuron.

[17] Götz J., Richter-Stretton G., Cruz E. (2021). Therapeutic Ultrasound as a Treatment Modality for Physiological and Pathological Ageing Including Alzheimer’s Disease. Pharmaceutics.

[18] Venturino A., Schulz R., De Jesus-Cortes H., Maes M.E., Nagy B., Reilly-Andujar F., Colombo G., Cubero R.J.A., Schoot Uiterkamp F.E., Bear M.F. (2021). Microglia enable mature perineuronal nets disassembly upon anesthetic ketamine exposure or 60-Hz light entrainment in the healthy brain. Cell Rep..

[19] Blackmore D.G., Turpin F., Palliyaguru T., Evans H.T., Chicoteau A., Lee W., Pelekanos M., Nguyen N., Song J., Sullivan R.K.P. (2021). Low-intensity ultrasound restores long-term potentiation and memory in senescent mice through pleiotropic mechanisms including NMDAR signaling. Mol. Psychiatry.

[20] Krasovitski B., Frenkel V., Shoham S., Kimmel E. (2011). Intramembrane cavitation as a unifying mechanism for ultrasound-induced bioeffects. Proc. Natl. Acad. Sci. USA.

[21] Gateau J., Aubry J.F., Chauvet D., Boch A.L., Fink M., Tanter M. (2011). In vivo bubble nucleation probability in sheep brain tissue. Phys. Med. Biol..

[22] Oh S.J., Lee J.M., Kim H.B., Lee J., Han S., Bae J.Y., Hong G.S., Koh W., Kwon J., Hwang E.S. (2020). Ultrasonic Neuromodulation via Astrocytic TRPA1. Curr. Biol..

[23] Yoo S., Mittelstein D.R., Hurt R.C., Lacroix J., Shapiro M.G. (2022). Focused ultrasound excites cortical neurons via mechanosensitive calcium accumulation and ion channel amplification. Nat. Commun..

[24] Nisbet R.M., van der Jeugd A., Leinenga G., Evans H.T., Janowicz P.W., Götz J. (2017). Combined effects of scanning ultrasound and a tau-specific single chain antibody in a tau transgenic mouse model. Brain.

[25] Lu R.M., Hwang Y.C., Liu I.J., Lee C.C., Tsai H.Z., Li H.J., Wu H.C. (2020). Development of therapeutic antibodies for the treatment of diseases. J. Biomed. Sci..

[26] Golde T.E. (2014). Open questions for Alzheimer’s disease immunotherapy. Alzheimers Res. Ther..

[27] Goedert M., Spillantini M.G. (2006). A century of Alzheimer’s disease. Science.

[28] Hardy J. (2006). Alzheimer’s disease: The amyloid cascade hypothesis: An update and reappraisal. J. Alzheimers Dis..

[29] Ittner L.M., Ke Y.D., Delerue F., Bi M., Gladbach A., van Eersel J., Wolfing H., Chieng B.C., Christie M.J., Napier I.A. (2010). Dendritic function of tau mediates amyloid-beta toxicity in Alzheimer’s disease mouse models. Cell.

[30] Ittner L.M., Götz J. (2011). Amyloid-beta and tau—A toxic pas de deux in Alzheimer’s disease. Nat. Rev. Neurosci..

[31] Götz J., Bodea L.G., Goedert M. (2018). Rodent models for Alzheimer disease. Nat. Rev. Neurosci..

[32] Jordao J.F., Thevenot E., Markham-Coultes K., Scarcelli T., Weng Y.Q., Xhima K., O’Reilly M., Huang Y., McLaurin J., Hynynen K. (2013). Amyloid-beta plaque reduction, endogenous antibody delivery and glial activation by brain-targeted, transcranial focused ultrasound. Exp. Neurol..

[33] Leinenga G., Götz J. (2015). Scanning ultrasound removes amyloid-beta and restores memory in an Alzheimer’s disease mouse model. Sci. Transl. Med..

[34] Leinenga G., Bodea L.G., Schröder J., Sun G., Zhou Y., Song J., Grubman A., Polo J.M., Götz J. (2021). Transcriptional signature in microglia isolated from an Alzheimer’s disease mouse model treated with scanning ultrasound. Bioeng. Transl. Med..

[35] Mathew A.S., Gorick C.M., Price R.J. (2021). Multiple regression analysis of a comprehensive transcriptomic data assembly elucidates mechanically- and biochemically-driven responses to focused ultrasound blood-brain barrier disruption. Theranostics.

[36] Poon C.T., Shah K., Lin C., Tse R., Kim K.K., Mooney S., Aubert I., Stefanovic B., Hynynen K. (2018). Time course of focused ultrasound effects on beta-amyloid plaque pathology in the TgCRND8 mouse model of Alzheimer’s disease. Sci. Rep..

[37] Poon C., Pellow C., Hynynen K. (2021). Neutrophil recruitment and leukocyte response following focused ultrasound and microbubble mediated blood-brain barrier treatments. Theranostics.

[38] Hsu P.H., Lin Y.T., Chung Y.H., Lin K.J., Yang L.Y., Yen T.C., Liu H.L. (2018). Focused Ultrasound-Induced Blood-Brain Barrier Opening Enhances GSK-3 Inhibitor Delivery for Amyloid-Beta Plaque Reduction. Sci. Rep..

[39] Xhima K., Markham-Coultes K., Kofoed R.H., Saragovi H.U., Hynynen K., Aubert I. (2021). Ultrasound delivery of a TrkA agonist confers neuroprotection to Alzheimer-associated pathologies. Brain.

[40] Deng Z., Wang J., Xiao Y., Li F., Niu L., Liu X., Meng L., Zheng H. (2021). Ultrasound-mediated augmented exosome release from astrocytes alleviates amyloid-beta-induced neurotoxicity. Theranostics.

[41] Chan T.G., Ruehl C.L., Morse S.V., Simon M., Rakers V., Watts H., Aprile F.A., Choi J.J., Vilar R. (2021). Modulation of amyloid-beta aggregation by metal complexes with a dual binding mode and their delivery across the blood-brain barrier using focused ultrasound. Chem. Sci..

[42] Zhu Q., Xu X., Chen B., Liao Y., Guan X., He Y., Cui H., Rong Y., Liu Z., Xu Y. (2022). Ultrasound-targeted microbubbles destruction assists dual delivery of beta-amyloid antibody and neural stem cells to restore neural function in transgenic mice of Alzheimer’s disease. Med. Phys..

[43] Leinenga G., Koh W.K., Götz J. (2019). Scanning ultrasound in the absence of blood-brain barrier opening is not sufficient to clear beta-amyloid plaques in the APP23 mouse model of Alzheimer’s disease. Brain Res. Bull..

[44] Eguchi K., Shindo T., Ito K., Ogata T., Kurosawa R., Kagaya Y., Monma Y., Ichijo S., Kasukabe S., Miyata S. (2018). Whole-brain low-intensity pulsed ultrasound therapy markedly improves cognitive dysfunctions in mouse models of dementia—Crucial roles of endothelial nitric oxide synthase. Brain Stimul..

[45] Karakatsani M.E., Kugelman T., Ji R., Murillo M., Wang S., Niimi Y., Small S.A., Duff K.E., Konofagou E.E. (2019). Unilateral Focused Ultrasound-Induced Blood-Brain Barrier Opening Reduces Phosphorylated Tau from The rTg4510 Mouse Model. Theranostics.

[46] Pandit R., Leinenga G., Götz J. (2019). Repeated ultrasound treatment of tau transgenic mice clears neuronal tau by autophagy and improves behavioral functions. Theranostics.

[47] Caballero B., Wang Y., Diaz A., Tasset I., Juste Y.R., Stiller B., Mandelkow E.M., Mandelkow E., Cuervo A.M. (2018). Interplay of pathogenic forms of human tau with different autophagic pathways. Aging Cell.

[48] Shen Y., Hua L., Yeh C.K., Shen L., Ying M., Zhang Z., Liu G., Li S., Chen S., Chen X. (2020). Ultrasound with microbubbles improves memory, ameliorates pathology and modulates hippocampal proteomic changes in a triple transgenic mouse model of Alzheimer’s disease. Theranostics.

[49] Janowicz P.W., Leinenga G., Götz J., Nisbet R.M. (2019). Ultrasound-mediated blood-brain barrier opening enhances delivery of therapeutically relevant formats of a tau-specific antibody. Sci. Rep..

[50] Jordao J.F., Ayala-Grosso C.A., Markham K., Huang Y., Chopra R., McLaurin J., Hynynen K., Aubert I. (2010). Antibodies targeted to the brain with image-guided focused ultrasound reduces amyloid-beta plaque load in the TgCRND8 mouse model of Alzheimer’s disease. PLoS ONE.

[51] Leinenga G., Koh W.K., Götz J. (2021). A comparative study of the effects of Aducanumab and scanning ultrasound on amyloid plaques and behavior in the APP23 mouse model of Alzheimer disease. Alzheimers Res. Ther..

[52] Sun T., Shi Q., Zhang Y., Power C., Hoesch C., Antonelli S., Schroeder M.K., Caldarone B.J., Taudte N., Schenk M. (2021). Focused ultrasound with anti-pGlu3 Abeta enhances efficacy in Alzheimer’s disease-like mice via recruitment of peripheral immune cells. J. Control. Release.

[53] Lipsman N., Meng Y., Bethune A.J., Huang Y., Lam B., Masellis M., Herrmann N., Heyn C., Aubert I., Boutet A. (2018). Blood-brain barrier opening in Alzheimer’s disease using MR-guided focused ultrasound. Nat. Commun..

[54] Park S.H., Baik K., Jeon S., Chang W.S., Ye B.S., Chang J.W. (2021). Extensive frontal focused ultrasound mediated blood-brain barrier opening for the treatment of Alzheimer’s disease: A proof-of-concept study. Transl. Neurodegener..

[55] Epelbaum S., Burgos N., Canney M., Matthews D., Houot M., Santin M.D., Desseaux C., Bouchoux G., Stroer S., Martin C. (2022). Pilot study of repeated blood-brain barrier disruption in patients with mild Alzheimer’s disease with an implantable ultrasound device. Alzheimers Res. Ther..

[56] Pouliopoulos A.N., Wu S.Y., Burgess M.T., Karakatsani M.E., Kamimura H.A.S., Konofagou E.E. (2020). A Clinical System for Non-invasive Blood-Brain Barrier Opening Using a Neuronavigation-Guided Single-Element Focused Ultrasound Transducer. Ultrasound Med. Biol..

[57] Turturro A., Duffy P., Hass B., Kodell R., Hart R. (2002). Survival characteristics and age-adjusted disease incidences in C57BL/6 mice fed a commonly used cereal-based diet modulated by dietary restriction. J. Gerontol. A Biol. Sci. Med. Sci..

[58] Lopez-Otin C., Blasco M.A., Partridge L., Serrano M., Kroemer G. (2013). The hallmarks of aging. Cell.

[59] Ghosh K., Agarwal P., Haggerty G. (2011). Alzheimer’s disease—Not an exaggeration of healthy aging. Indian J. Psychol. Med..

[60] Pievani M., Agosta F., Pagani E., Canu E., Sala S., Absinta M., Geroldi C., Ganzola R., Frisoni G.B., Filippi M. (2010). Assessment of white matter tract damage in mild cognitive impairment and Alzheimer’s disease. Hum. Brain Mapp..

[61] Fjell A.M., Amlien I.K., Westlye L.T., Stenset V., Fladby T., Skinningsrud A., Eilsertsen D.E., Bjornerud A., Walhovd K.B. (2010). CSF biomarker pathology correlates with a medial temporo-parietal network affected by very mild to moderate Alzheimer’s disease but not a fronto-striatal network affected by healthy aging. NeuroImage.

[62] Irwin K., Sexton C., Daniel T., Lawlor B., Naci L. (2018). Healthy Aging and Dementia: Two Roads Diverging in Midlife?. Front. Aging Neurosci..

[63] Willis E.F., Bartlett P.F., Vukovic J. (2017). Protocol for Short- and Longer-term Spatial Learning and Memory in Mice. Front. Behav. Neurosci..

[64] Benice T.S., Rizk A., Kohama S., Pfankuch T., Raber J. (2006). Sex-differences in age-related cognitive decline in C57BL/6J mice associated with increased brain microtubule-associated protein 2 and synaptophysin immunoreactivity. Neuroscience.

[65] van Praag H., Shubert T., Zhao C., Gage F.H. (2005). Exercise enhances learning and hippocampal neurogenesis in aged mice. J. Neurosci..

[66] Balbi M., Ghosh M., Longden T.A., Jativa Vega M., Gesierich B., Hellal F., Lourbopoulos A., Nelson M.T., Plesnila N. (2015). Dysfunction of mouse cerebral arteries during early aging. J. Cereb. Blood Flow Metab..

[67] Hatch R.J., Leinenga G., Götz J. (2016). Scanning ultrasound (SUS) causes no changes to neuronal excitability and prevents age-related reductions in hippocampal CA1 dendritic structure in wild-type mice. PLoS ONE.

[68] Blackmore D.G., Turpin F., Mohamed A.Z., Zong F., Pandit R., Pelekanos M., Nasrallah F., Sah P., Bartlett P.F., Götz J. (2018). Multimodal analysis of aged wild-type mice exposed to repeated scanning ultrasound treatments demonstrates long-term safety. Theranostics.

[69] Scarcelli T., Jordao J.F., O’Reilly M.A., Ellens N., Hynynen K., Aubert I. (2014). Stimulation of hippocampal neurogenesis by transcranial focused ultrasound and microbubbles in adult mice. Brain Stimul..

[70] Huang X., Lin Z., Wang K., Liu X., Zhou W., Meng L., Huang J., Yuan K., Niu L., Zheng H. (2019). Transcranial Low-Intensity Pulsed Ultrasound Modulates Structural and Functional Synaptic Plasticity in Rat Hippocampus. IEEE Trans. Ultrason. Ferroelectr. Freq. Control.

[71] Tyler W.J., Tufail Y., Finsterwald M., Tauchmann M.L., Olson E.J., Majestic C. (2008). Remote excitation of neuronal circuits using low-intensity, low-frequency ultrasound. PLoS ONE.

[72] Cheng Z., Wang C., Wei B., Gan W., Zhou Q., Cui M. (2022). High resolution ultrasonic neural modulation observed via in vivo two-photon calcium imaging. Brain Stimul..

[73] Beisteiner R., Matt E., Fan C., Baldysiak H., Schonfeld M., Philippi Novak T., Amini A., Aslan T., Reinecke R., Lehrner J. (2020). Transcranial Pulse Stimulation with Ultrasound in Alzheimer’s Disease-A New Navigated Focal Brain Therapy. Adv. Sci..

[74] Popescu T., Pernet C., Beisteiner R. (2021). Transcranial ultrasound pulse stimulation reduces cortical atrophy in Alzheimer’s patients: A follow-up study. Alzheimers Dement..

[75] Mueller J., Legon W., Opitz A., Sato T.F., Tyler W.J. (2014). Transcranial focused ultrasound modulates intrinsic and evoked EEG dynamics. Brain Stimul..

[76] Legon W., Sato T.F., Opitz A., Mueller J., Barbour A., Williams A., Tyler W.J. (2014). Transcranial focused ultrasound modulates the activity of primary somatosensory cortex in humans. Nat. Neurosci..

[77] Lee W., Kim H., Jung Y., Song I.U., Chung Y.A., Yoo S.S. (2015). Image-guided transcranial focused ultrasound stimulates human primary somatosensory cortex. Sci. Rep..

[78] Lee W., Chung Y.A., Jung Y., Song I.U., Yoo S.S. (2016). Simultaneous acoustic stimulation of human primary and secondary somatosensory cortices using transcranial focused ultrasound. BMC Neurosci..

[79] Monti M.M., Schnakers C., Korb A.S., Bystritsky A., Vespa P.M. (2016). Non-Invasive Ultrasonic Thalamic Stimulation in Disorders of Consciousness after Severe Brain Injury: A First-in-Man Report. Brain Stimul..

[80] Lee W., Kim H.C., Jung Y., Chung Y.A., Song I.U., Lee J.H., Yoo S.S. (2016). Transcranial focused ultrasound stimulation of human primary visual cortex. Sci. Rep..

[81] Lee W., Kim S., Kim B., Lee C., Chung Y.A., Kim L., Yoo S.S. (2017). Non-invasive transmission of sensorimotor information in humans using an EEG/focused ultrasound brain-to-brain interface. PLoS ONE.

[82] Ai L., Bansal P., Mueller J.K., Legon W. (2018). Effects of transcranial focused ultrasound on human primary motor cortex using 7T fMRI: A pilot study. BMC Neurosci..

[83] Legon W., Bansal P., Tyshynsky R., Ai L., Mueller J.K. (2018). Transcranial focused ultrasound neuromodulation of the human primary motor cortex. Sci. Rep..

[84] Legon W., Ai L., Bansal P., Mueller J.K. (2018). Neuromodulation with single-element transcranial focused ultrasound in human thalamus. Hum. Brain Mapp..

[85] Brinker S.T., Preiswerk F., White P.J., Mariano T.Y., McDannold N.J., Bubrick E.J. (2020). Focused Ultrasound Platform for Investigating Therapeutic Neuromodulation Across the Human Hippocampus. Ultrasound Med. Biol..

[86] Fomenko A., Chen K.S., Nankoo J.F., Saravanamuttu J., Wang Y., El-Baba M., Xia X., Seerala S.S., Hynynen K., Lozano A.M. (2020). Systematic examination of low-intensity ultrasound parameters on human motor cortex excitability and behavior. eLife.

[87] Badran B.W., Caulfield K.A., Stomberg-Firestein S., Summers P.M., Dowdle L.T., Savoca M., Li X., Austelle C.W., Short E.B., Borckardt J.J. (2020). Sonication of the anterior thalamus with MRI-Guided transcranial focused ultrasound (tFUS) alters pain thresholds in healthy adults: A double-blind, sham-controlled study. Brain Stimul..

[88] Sanguinetti J.L., Hameroff S., Smith E.E., Sato T., Daft C.M.W., Tyler W.J., Allen J.J.B. (2020). Transcranial Focused Ultrasound to the Right Prefrontal Cortex Improves Mood and Alters Functional Connectivity in Humans. Front. Hum. Neurosci..

[89] Braun V., Blackmore J., Cleveland R.O., Butler C.R. (2020). Transcranial ultrasound stimulation in humans is associated with an auditory confound that can be effectively masked. Brain Stimul..

[90] Stern J.M., Spivak N.M., Becerra S.A., Kuhn T.P., Korb A.S., Kronemyer D., Khanlou N., Reyes S.D., Monti M.M., Schnakers C. (2021). Safety of focused ultrasound neuromodulation in humans with temporal lobe epilepsy. Brain Stimul..

[91] Kamimura H.A.S., Conti A., Toschi N., Konofagou E.E. (2020). Ultrasound neuromodulation: Mechanisms and the potential of multimodal stimulation for neuronal function assessment. Front. Phys..

[92] Dauba A., Delalande A., Kamimura H.A.S., Conti A., Larrat B., Tsapis N., Novell A. (2020). Recent Advances on Ultrasound Contrast Agents for Blood-Brain Barrier Opening with Focused Ultrasound. Pharmaceutics.

[93] Padilla F., Ter Haar G. (2022). Recommendations for Reporting Therapeutic Ultrasound Treatment Parameters. Ultrasound Med. Biol..

